# Downregulation of miR-21 gene expression by CRE-Ter to modulate osteoclastogenesis: De Novo mechanism

**DOI:** 10.1016/j.bbrep.2021.101002

**Published:** 2021-04-23

**Authors:** Yutthana Pengjam, Thanet Prajantasen, Natda Tonwong, Pharkphoom Panichayupakaranant

**Affiliations:** a Faculty of Medical Technology, Prince of Songkla University, Songkhla, 90110, Thailand; b Phytomedicine and Pharmaceutical Biotechnology Excellent Center (PPBEC), Faculty of Pharmaceutical Sciences Prince of Songkla University, Songkhla, 90110, Thailand

**Keywords:** CRE-Ter, miR-21, Osteoclastogenesis, NFκB, Akt

## Abstract

miR-21 expression stimulates osteoclast cells in the context of osteoclastogenesis. A previous report showed that NFκB-miR-21 pathway could serve as an innovative alternative to devise therapeutics for healing diabetic ulcers. Furthermore, our study demonstrated that a highly water-soluble curcuminoids-rich extract (CRE-Ter) inhibits osteoclastogenesis through NFκB pathway. The interplay between miR-21 and CRE-Ter in osteoclastogenesis has not yet been investigated. In this study, we examined the relation of CRE-Ter and miR-21 gene expression in receptor of the nuclear factor κB (NFκB) ligand (RANKL) - induced murine monocyte/macrophage RAW 264.7 cells, osteoclast cells, in osteoclastogenesis. Effect of CRE-Ter on generation of intracellular reactive oxygen species (ROS) was estimated by dichlorofluorescein diacetate (DCFH-DA). The results reveal that CRE-Ter reduced expression levels of miR-21 gene in osteoclasts. The inhibitory effects of CRE-Ter on *in vitro* osteoclastogenesis were evaluated by reduction in tartrate-resistant acid phosphatase (TRAP) content, and by reduction in expression levels of an osteoclast-specific gene, cathepsin K. Treatment of the osteoclast cells with CRE-Ter suppressed RANKL-induced NFκB activation including phospho-NFκB-p65, and phospho IκBα proteins. Western blot analysis revealed that NFκB inhibitor up-regulated CRE-Ter-promoted expression of phospho-NFκB-p65. In addition, CRE-Ter dose-dependently inhibited phospho-Akt expression. CRE-Ter also dose-dependently reduced DNA binding activity of NFκB and Akt as revealed by EMSA. ChIP assay revealed binding of NFκB-p65 to miR-21 promoters. In conclusion, our results demonstrate that CRE-Ter downregulates miR-21 gene expression in osteoclasts via a de novo mechanism, NFκB- Akt-miR-21 pathway.

## 1 Introduction

Osteoclasts, the exclusive bone resorptive cells, are derived from hematopoietic stem cells through the common myeloid progenitor to the colony-forming unit from macrophage, then become an osteoclast lineage, and receptor of the nuclear factor κB (NFκB) ligand (RANKL) - induced murine monocyte/macrophage RAW 264.7 cells [1,2]. Osteoclast differentiation, function, and survival are regulated by several exogenous cytokines such as macrophage colony stimulating factor (M-CSF), receptor activation, NFκB ligand (RANKL), tumor necrosis factor α, interleukin-1 and interleukin-6 [3,4]. Osteoclast precursors that express RANK, a tumor necrosis factor (TNF) receptor family member, recognize RANKL and differentiate into osteoclasts in the presence of macrophage/monocyte colony stimulating factor (M-CSF) [5]. RANK-RANKL interaction is essential to activate a variety of downstream signaling pathways required for osteoclast development [6]. The interaction of RANKL with RANK results in a cascade of intercellular events including the expression of NFκB, Akt, MAPKs, nuclear factor of activated T cells (NFAT), and ionic calcium and calcium/calmodulin-dependent kinase [[7], [8], [9], [10], [11]]. The final process of differentiation is characterized by acquisition of mature phenotypic osteoclast markers, such as expression of tartrate-resistant acid phosphatase (TRAP), cathepsin K, matrix metalloproteinase 9 (MMP-9) as well as morphological conversion into large multinucleated cells and the ability to form resorption lacunae in bones [12,13].

MicroRNAs (miRs), which regulate gene expression, are transcribed as a primary miRs (pri-miRs) containing a 5′- cap structure and poly (A) tail that are processed to produce the mature miRs. Two nuclease enzymes, the nuclear RNase III Drosha and the cytosolic RNaseIII Dicer, are known to act sequentially to trim the miRs to mature form. Hundreds of miR genes have been identified in the human genome, and it is estimated that one-third of protein-coding genes are regulated by miRs. Hence, miRs constitute one of the most abundant classes of gene-regulatory molecules in an animal, and they are implicated in almost every biological process, including development timing, cell differentiation, cell proliferation, cell death, metabolic control, transposon silencing, and antiviral defense [14,15].

Curcuminoids are hydrophobic polyphenolic compounds, obtained from the rhizome of the turmeric herb (*Curcuma longa*) [16]. Curcuminoids, which include curcumin (Cu), demethoxycurcumin (De) and bis-demethoxycurcumin (Bis), have been described previously as major active components of the turmeric herb [17,18]. Currently they are not approved as medicinal agents because of the limited solubility in aqueous environments, for example in the human gastrointestinal tract [17,18], and the rapid metabolism both in intestine and in liver [19]. Curcuminoids are mostly consumed in Southeast Asian countries as culinary spices and have been used in Indian Ayurvedic medicine for centuries. Current trends in scientific research have shown that curcuminoids have efficacy toward arthritis, diabetes, multiple sclerosis, common cold, inflammation, and indigestion [[20], [21], [22], [23], [24]]. They have also shown significant *in vitro* and *in vivo* preventive activities against various forms of cancer, including leukemia, melanoma, lymphoma, breast cancer, ovarian cancer, and lung cancer [[25], [26], [27], [28]]. Because of their limited aqueous solubility, a highly water-soluble curcuminoids-rich extract, CRE-Ter (CRE), was first dispersed in polyvinylpyrrolidone K30 (PVP K30), then incorporated with hydroxypropyl-β-cyclodextrin (HPBCD). CRE-Ter is more soluble than its major active compounds [29,30].

Although many recent studies have showed that more than the 12 miRs miR-26a, miR-125b, miR-133, miR-135, miR-29a, miR-141, miR-200a, miR-210, miR-29, miR-378, miR-2861, and miR-206, are implicated in osteoblast differentiation [15], the function of miRs on the context of osteoclastogensis is, however, poorly understood [31]. Previous reports have shown that the microRNA miR-21 is upregulated during RANKL-induced osteoclastogenesis [31]. On the other hand, a previous report has shown that NFκB-miR-21 pathway could serve as an innovative target to develop therapeutics for healing diabetic ulcers. NFκB activation is necessary for miR-21 activation in fibroblast cells [32]. In addition, our current studies in the context of bone resorption show that the highly water-soluble curcuminoids-rich extract (CRE-Ter) inhibits osteoclastogenesis through NFκB pathway. The interplay between miR-21 and CRE-Ter in the context of osteoclastogenesis has not yet been investigated. In this study, we examined the relation of CRE-Ter and miR-21 gene in receptor of the nuclear factor κB (NFκB) ligand (RANKL) - induced murine monocyte/macrophage RAW 264.7 cells, osteoclast cells, in the context of osteoclastogenesis.

## 2 Materials and methods

### 2.1 Reagents

CRE-Ter (containing 14% w/w curcuminoids) was made using methods previously described [29,30]. RANKL was purchased from R&D system (Minneapolis, USA). DMEM with 10% heat inactivated FBS, glutamine and anti-bacterial cocktail were purchased from Gibco Co. (Tokyo, Japan). Leukocyte acid phosphatase assay kit for TRAP staining was purchased from Sigma (St. Louis, MO, USA). Specific antibodies, anti Akt, anti phospho-Akt, anti NFκB-p65, anti phospho-NFκB-p65, anti IκBα, anti phospho IκBα, and anti β-actin, were purchased from Cell Signaling Technology (Beverly, MA, USA). RNA extraction kit (RNease kit) and miRNeasy (Qiagen) were purchased from Qiagen (Valencia, CA. USA). All other reagents were from Sigma Chemical Co. or Wako Pure Chemical Industries Ltd, Japan.

### 2.2 CRE-Ter preparation

**Fig. 1:**
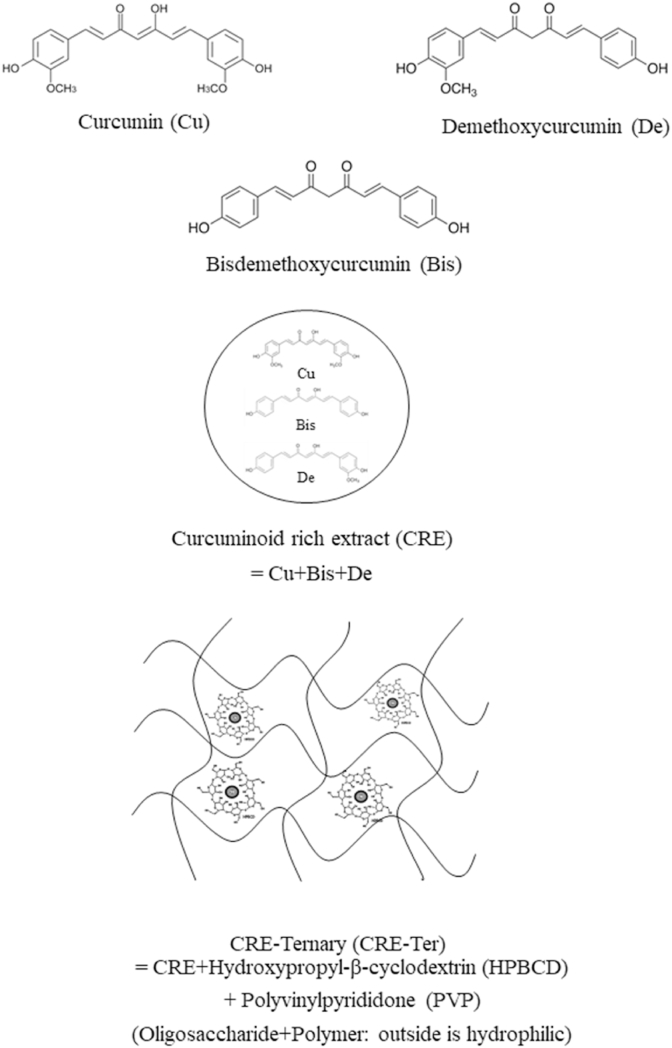
Chemical structure of CRE-Ter.

CRE-Ter (containing 14% w/w curcuminoids) was made using methods previously described [29,30]. Briefly, dried turmeric powder was extracted with ethanol using microwave-assisted extraction in the following conditions: power of 900 Watt, at 70–75 °C, with three irradiation cycles (one cycle = 3 min power-on and 30 s power-off). The extract was filtered and then eluted in a Diaion® HP-20 column with 55% and 60% v/v ethanol, respectively, to obtain curcuminoids enriched extract (CRE). CRE-Ter was prepared using solvent evaporation. Firstly, CRE was dispersed in polyvinylpyrrolidone K30 (PVP K30) (9% w/w), then incorporated with hydroxypropyl-β-cyclodextrin (HPBCD) in 1:1 M ratio and subsequently solvent evaporated under reduced pressure to obtain CRE-Ter powder. Based on HPLC analysis, CRE-SD contained 14% w/w curcuminoids (Fig. 1).

### 2.3 Cell cultures

Mouse monocyte/macrophage RAW 264.7 cells purchased from the RIKEN Cell Bank (Tsukuba, Japan) were grown in DMEM containing 10% heat inactivated FBS, 2 mM glutamine, 100 U/ml penicillin G, and 100 μg/ml streptomycin sulfate in a humidified chamber (5% CO_2_, 37 °C). For osteoclast differentiation, RAW 264.7 cells were suspended in DMEM containing 10% heat inactivated FBS, 2 mM glutamine, 100 U/ml penicillin G, and 100 μg/ml streptomycin sulfate, seeded at 3 × 10^3^ cells/well in 96-well culture plates and cultured with 20 ng/ml soluble RANKL for 5 days.

### 2.4 Osteoclastogenesis confirmation test by TRAP assay

RAW 264.7 cells with RANKL (20 ng/ml) and CRE-Ter (0, 5, 10, 20 or 30 μg/ml) were cultured separately in 12 well culture vessel in DMEM containing 10% heat inactivated FBS, 2 mM glutamine, 100 U/ml penicillin G, and 100 μg/ml streptomycin sulfate for 5 days in a humidified chamber (5% CO_2_, 37 °C). At the end of this time period, TRAP assay was conducted using TRAP assay kit (TaKaRa, Bio, Inc, Tokyo, Japan) according to the manufacturer's instructions. TRAP-positive multinucleated cells containing dark orange cells were monitored under a light microscope (Nikon, model # MFA20100, Japan).

### 2.5 RT-PCR and real –time RT-PCR

RAW 264.7 cells were treated with RANKL (20 ng/ml) and CRE-Ter (0, 5, 10, 20 or 30 μg/ml) and incubated for 5 days in standard conditions of humidified chamber (5% CO_2_, 37 °C). MiRNA enriched total RNA was isolated using miRNeasy kit (Qiagen, Japan). One microgram miRNA enriched total RNA was used to synthesize first strand cDNA by using miScript II RT kit (Qiagen). The first strand cDNA was further used in conjunction with miScript Primer assays and SYBR Green PCR kit (Qiagen) for analyses of primary, precursor and mature forms of miR-21 gene by RT-PCR and Real-time RT-PCR respectively, using ABI Prism 7500 HT sequence detection system (Applied Biosystems, CA, USA).

### 2.6 Osteoclastogenesis confirmation test by RT-PCR of cathepsin K

RAW 264.7 cells were treated with RANKL (20 ng/ml) and CRE-Ter (0, 5, 10, 20 or 30 μg/ml) and incubated for 5 days, in a humidified chamber (5% CO_2_, 37 °C). Total RNA was isolated using ISOGEN (Nippon Gene, Toyama, Japan) and cDNA was synthesized using ReverTra Ace qPCR kit (Toyobo, Osaka, Japan), according to the manufacturer's instructions. RT- PCR of cathepsin K mRNA was performed with FastStart SYBR Green Master mix (Roche Diagnostic, Mannheim, Germany) in Gene Atlas thermocycler (Astec Co, Japan).

### 2.7 Western blotting

RAW 264.7 cells were treated with RANKL (20 ng/ml) and CRE-Ter (0, 5, 10, 20 or 30 μg/ml) and incubated for 5 days in standard conditions of humidified chamber (5% CO_2_, 37 °C). After incubation period, cell lysates were obtained by standard methods. Cell lysates were re-suspended in sodium dodecyl sulfate-polyacrylamide gel electrophoresis (SDS-PAGE) buffer containing 2-mercatoethanol, and boiled at 95 °C for 5 min. Protein samples were subjected to SDS-PAGE in a 10% polyacrylamide gel, and subsequently electroblotted onto polyvinylidene fluoride (PVDF) membranes (GE Healthcare, NJ, USA). After blocking of non-specific binding sites in 3% nonfat milk in TPBS (PBS and 0.1% Tween 20), the membranes were incubated over-night at 4 °C with specific primary antibodies. The membranes were washed in TPBS and incubated further with horse-radish peroxidase-conjugated secondary antibodies at room temperature. Protein bands were detected using an enhanced ECL kit (GE Healthcare, Japan) and LAS4000 chemiluminescence detector.

### 2.8 Reactive oxygen species assay

Effect of CRE-Ter on generation of intracellular reactive oxygen species (ROS) was estimated by dichlorofluorescein diacetate (DCFH-DA), using Oxiselect™ Intracellular ROS assay kit (Cell Bio Lab, Inc., San Diego, CA 92126 USA). Following the incubation of RANKL stimulated RAW 264.7 cells with various doses of CRE-Ter (0, 5, 10, 20 or 30 μg/ml) for 12 h, 10 μmol/L DCFH-DA was added to cells. Non-fluorescent DCFH is converted to fluorescent DCFH-DA in proportion to the amount of generated intracellular ROS generation. The fluorescence was measured using spectrofluorometer (Beckman Coulter, Inc, CA, USA) at excitation and emission of 485 and 530 nm respectively.

### 2.9 Electrophoretic mobility shift assay (EMSA) for NFκB

RAW 264.7 cells were treated with RANKL (20 ng/ml) and CRE-Ter (0, 10, or 20 μg/ml) and incubated for 5 days in standard conditions of humidified chamber (5% CO_2_, 37 °C). Nuclear extracts were prepared and EMSA was carried out as described previously [33]. Briefly, equal amounts of nuclear extracts from untreated and RANKL-treated cells were incubated with biotin labelled oligonucleotides (15 μg protein with 15 fmol DNA), NFκB (Forward): 5′-AGTTGAGGGGACTTTCCCAGGC-3′ and NFκB (Reverse): 5′-GCCTGGGAAAGTCCCCTCAACT-3′ for 30 min at 37 °C. The DNA-protein complex formed was separated from free oligonucleotides on 5% native polyacrylamide gels and subsequently electroblotted onto polyvinylidene fluoride (PVDF) membranes (GE Healthcare, NJ, USA). The DNA-protein complex was cross-linked to the membrane by baking in incubator for 2 h at 80 °C. After blocking of non-specific binding sites with blocking buffer, the membrane was incubated with stabilized streptavidin-horseradish peroxidase-conjugate, washed and equilibrated with substrate equilibrium buffer. The membrane was finally incubated with luminol/enhancer solution and stable peroxide solution. DNA-protein band complex was detected using LAS4000 chemiluminescence detector (Fujifilm, Japan).

### 2.10 Chromatin immunoprecipitation (CHIP) assay

RAW264.7 cells with RANKL (20 ng/ml) and CRE-Ter (0, 10, or 20 μg/ml) were cultured separately in 12 well culture vessel and incubated for 5 days in standard conditions of humidified chamber (5% CO_2_, 37 °C). Cells were cross-linked for 10 min with 1% formaldehyde, and chromatin was isolated using Pierce™ Chromatin Prep module (Thermo Scientific). The chromatin was subjected to immunoprecipitation with anti- NFκB p65 antibody or IgG as a negative control, using Pierce™ agarose ChIP kit (Thermo Scientific). After stringent washing, DNA was eluted and subjected to PCR using pri-miR-21 primers encompassing NFκB binding sites (set A: Forward 5′- GGAGTGGATGGGTTCTGCCTTA - 3′ and Reverse 5′- CAAGGTGGATTGCATCGAGG -3′ set B: Forward 5′- TGCAACAGACTGGCCTTC-3′ and Reverse 5′- CATGCAAGACTGTTATCCAATCT-3′

### 2.11 Treatment with NFκB inhibitor

Subconfluent cells were treated with JSH 23, NFκB transcriptional activity (IC50 = 7.1 μM), for 4 h, followed by 16 h incubation with CRE-Ter 20 μg/ml. After incubation period, cell lysates were obtained by standard methods. Cell lysates were used for Western blot detection of phospho-NFκB-p65 and NFκB-p65. β actin was used as a housekeeping control.

### 2.12 Statistical analysis

All data are expressed as the mean ± SD. Statistical analyses of the significance of differences among values were carried out by one-way ANOVA with a *post hoc* Dunnett's test or Student's t-test. Values of P < 0.05 were considered to indicate statistical significance.

## 3 Results

### 3.1 CRE-Ter prevents osteoclast differentiation

**Fig. 2:**
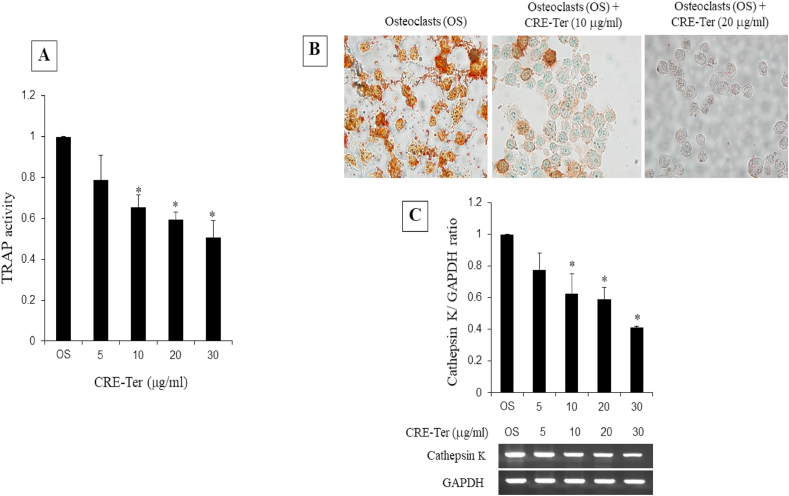
Inhibition of Osteoclastogenesis by CRE-Ter. (2A) Values are percentages of TRAP content in cell. 2B) Osteoclast marker protein TRAP was measured for 5 days. Whole cell TRAP staining was done according to standard procedure and cells were photographed in 100 × bright field microscopy. (2C) Semi-quantitative RT-PCR assay of cathepsin K gene. GAPDH gene expression was used as internal control. mRNA expression is expressed as mean ratio between cathepsin K and GAPDH. The representative images were selected as representative data from three independent experiments and data represent mean ± S.D. of three independent experiments. P < 0.05 (*) indicates statistical significance with control (OS).

Our recent studies in the context of bone resorption show that CRE-Ter inhibits RANKL-induced differentiation of RAW 264.7 cells into osteoclasts. RAW 264.7 cell differentiation to osteoclast is characterized by acquisition of mature phenotypic osteoclast markers such as expression of tartrate-resistant acid phosphatase (TRAP), cathepsin K, as well as morphological conversion into large multinucleated cells [12]. Preliminary studies were conducted to analyze the effects of CRE-Ter on the viability or proliferation of osteoclasts. CRE-Ter in dose-dependent manner suppressed *in vitro* osteoclastogenesis by reduction in tartrate-resistant acid phosphatase (TRAP) content (Fig. 2A). Exposure of osteoclast cells to CRE-Ter in two doses, 10 and 20 μg/ml resulted in reducing the number of TRAP-positive cells (Fig. 2B). In addition, CRE-Ter dose-dependently reduced cathepsin K expression (Fig. 2C). The results thus far strengthen the concept that CRE-Ter prevents differentiation of osteoclasts.

### 3.2 Downregulation of miR-21 gene expression in osteoclast by CRE-Ter

**Fig. 3:**
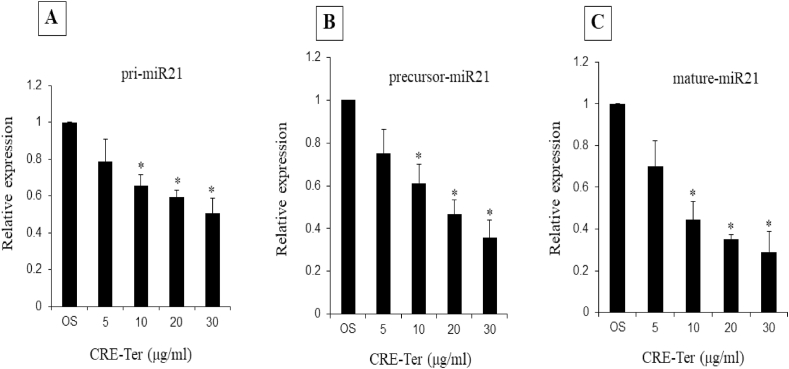
Downregulation of miR-21 gene expression in osteoclast by CRE-Ter. The primary (Pri miR-21, A), precursor (Pre miR-21, B) and Mature miR-21 (C) forms of miR-21 were determined by Real-time RT-PCR. The amount of miR-21 was obtained by normalizing to the level of SnRNA U6 in the samples. The data represent mean ± S.D. of three independent experiments. P < 0.05 (*) indicates statistical significance with control (OS).

Osteoclasts were treated with CRE-Ter at various doses (0, 5, 10, 20 or 30 μg/ml), and analyzed for expression of primary, precursor, and mature forms of miR21 gene. CRE-Ter downregulated all three forms of miR-21 gene expression. The primary (A), precursor (B) and mature forms (C) of miR-21 gene expression were significantly reduced in the presence of CRE-Ter (Fig. 3).

CRE-Ter prevents Akt and NFκB signaling molecule activation and interrelationship between ROS upon CRE-Ter treatment.

**Fig. 4:**
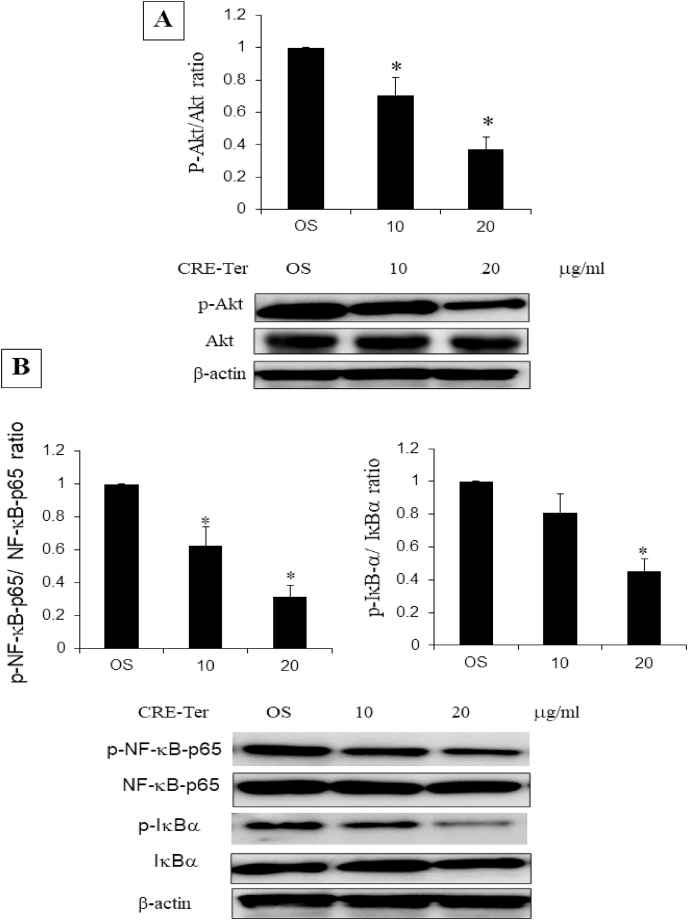
Effect of CRE-Ter on Akt family up-stream pathway (4A) and NFκB family (4B). WB analysis was done for Akt family and NFκB family. β-actin was used as housekeeping protein. The representative images were selected as representative data from three independent experiments and data represent mean ± S.D. of three independent experiments. P < 0.05 (*) indicates statistical significance with control (OS).

**Fig. 5:**
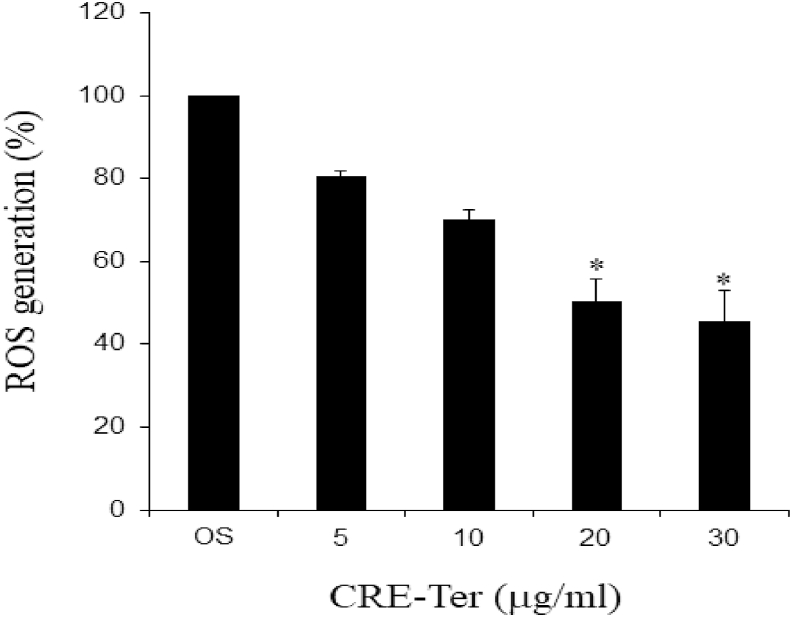
ROS generation assay Raw 264.7 cells were treated with RANKL (20 ng/ml) and different concentration of CRE-Ter for 12 h. Percentage of ROS generation was measured in culture medium and expressed. The data represent mean ± S.D. of three independent experiments. P < 0.05 (*) indicates statistical significance with control (OS).

**Fig. 6:**
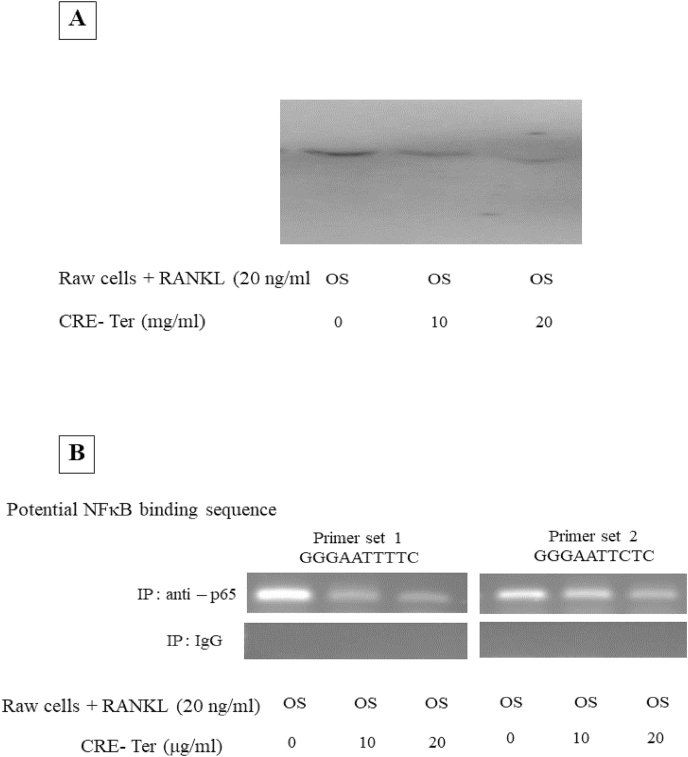
NF-κB activity by EMSA assay (6A). RANKL induced NF-κB activation and inhibitory action of CRE-Ter was dose dependent. (6B) ChIP was performed with anti p65 antibody or non-specific IgG (control), followed by PCR amplification with two sets of miR-21 primers that encompass NFκB p65 binding sites. Representative of three independent experiments is shown. The representative images were selected as representative data from three independent experiments and data represent mean ± S.D. of three independent experiments.

Akt axis plays an important role in osteoclast differentiation upon RANKL stimulation [1,10] and activates downstream NFκB family. CRE-Ter in dose dependent manner inhibited phospho-Akt expression (Fig. 4A). In RANKL-induced osteoclast differentiation, RANKL/RANK signaling induces the activation of NFκB and mitogen-activated protein kinases (MAPKs), followed by c-Fos expression [1,[7], [8], [9], [10]]. The NFκB activating pathway that leads to phosphorylation of inhibitory protein IκBα and nuclear translocation of mostly p65-containing heterodimers is activated by a proinflammatory stimulus [1]. To assess the role of NFκB activation, we analyzed the expression of p65, phospho-p65 subunits, the inhibitor IκBα and phospho-IκBα. Treatment of the osteoclast cells with CRE-Ter suppressed RANKL-induced NFκB activation including phospho-NFκB-p65, and phospho-IκBα proteins in a significant manner (p < 0.05) (Fig. 4B). These data reveal that CRE-Ter caused degradation of the IκBα, and thus mediated the release and nuclear translocation of p65 subunit. In addition, owing to its anti-oxidant properties, curcumin suppresses osteoclast differentiation by scavenging the generated intracellular ROS, which acts as a secondary messenger in the RANKL-induced osteoclast differentiation signaling pathways [34]. NFκB and Akt axis play an important role in osteoclast differentiation upon RANKL stimulation [1,10]. This effect attenuates ROS-induced NFκB and Akt signaling pathways for osteoclastogenesis and then causes a decrease in NFATc1 and cathepsin K gene expression, which are the master genes involved in the osteoclastic differentiation [34]. The present study demonstrated that CRE-Ter dose-dependently reduced cathepsin K expression. In order to check the role of antioxidative phenomenon of CRE-Ter, the curcumin derivative, we examined the anti-oxidative role of CRE-Ter with different concentrations. Results indicated that CRE-Ter dose dependently reduced the intracellular ROS activity (Fig. 5). We believe that the inhibitory effect of CRE-Ter on osteoclastic differentiation via NFκB and Akt pathways may be associated with its anti-oxidative properties. Further evidence that CRE-Ter reduces DNA binding activity of NFκB was obtained by EMSA (Fig. 6A). Two potential NFκB binding sites have been reported in the promoter region of miR-21 gene [35]. In order to investigate direct interaction between miR-21 and NFκB, ChIP assay was performed using NFκB p65 antibody and specific miR-21 primers that encompass potential NFκB binding sites. CRE-Ter treatment caused significantly decreased the binding of p65 to both binding sites in miR-21 promoter; this was attenuated by NFI (Fig. 6B). These results show that CRE-Ter was instrumental in activating NFκB pathway and mediating miR-21 downregulation in osteoclast cells.

### 3.3 Involvement of signaling pathways

**Fig. 7:**
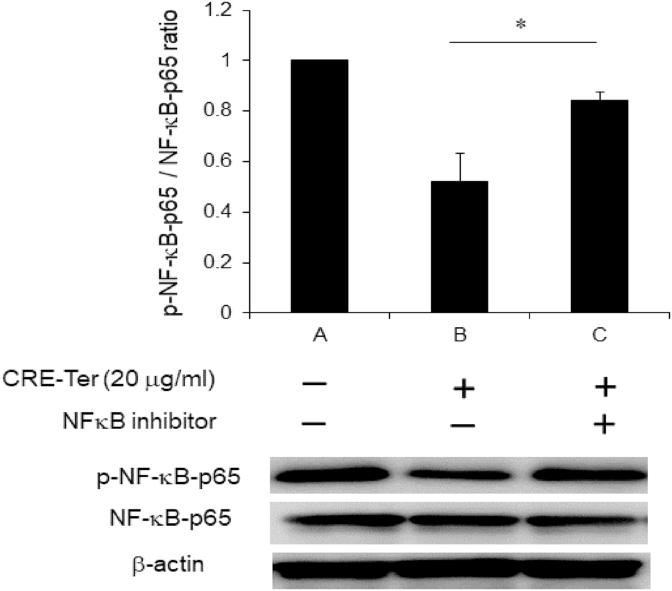
The influence of CRE-Ter was up-regulated in the presence of NFκB inhibitor. Cells were treated with CRE-Ter (20 μg/ml) in the presence of NFκB inhibitor, JSH 23, (IC50 = 7.1 μM). Protein expressions of phospho-NFκB-p65 and NFκB-p65 were assessed by Western blot analysis. The data represent mean ± S.D. of three independent experiments. P < 0.05 (*) indicates statistical significance with control (OS).

Members of the NFκB signaling pathway (phospho-NFκB-p65, and NFκB-p65 proteins) were studied to elucidate the possible participation of NFκB signaling pathways in the effects of CRE-Ter. Treatment with CRE-Ter caused a significant decrease in the activation of phospho-NFκB-p65. To test the involvement of signaling pathway, cells were treated with CRE-Ter in the presence of NFκB inhibitor (JSH 23). Co-treatment with the inhibitor up-regulated CRE-Ter-promoted expression of phospho-NFκB-p65, (Fig. 7), confirming the involvement miR-21 expression via NFκB signaling pathway.

## 4 Discussion

Osteoclasts are thought to arise both from the common myeloid progenitors (CMPs) through macrophage/dendritic cell progenitors or granulocyte/macrophage progenitors and receptor of the NFκB ligand (RANKL) - induced murine monocyte/macrophage RAW 264.7 cells [1,2]. Among the miRNAs studied, miR-21 showed a unique signature with increased expression, and it is associated with pathological conditions [36] including cancer [37], cardiac ischemia [38], and tissue fibrosis [39]. In addition, a previous report has shown that the miR-21 is up-regulated during RANKL-induced osteoclastogenesis. miR-21 was identified as miR expression signature of RANKL-induced osteoclastogenesis [31]. A previous report has shown that NFκB-miR-21 pathway could serve as an innovative target of therapeutics for healing diabetic ulcers. NFκB activation is necessary for miR-21 activation on fibroblast cells [32]. However, their interaction in osteoclast cells has not yet been investigated. In addition, our study in the context of bone resorption has shown that CRE-Ter in μg/ml levels inhibit osteoclastogenesis. The inhibitory effects of CRE-Ter against *in vitro* osteoclastogenesis were evaluated by reduction in tartrate-resistant acid phosphatase (TRAP) content, prevention of multinuclear formation in RAW cells, and were corroborated by significant reduction in expression levels of osteoclast-specific gene, cathepsin K. Treatment of the RAW 264.7 cells with CRE-Ter suppressed RANKL-induced NFκB activation with inhibition of Phospho-NFκB-p65, and Phospho- IκBα. In addition, CRE-Ter in a dose dependent manner inhibited phospho-Akt expression. Akt axis plays an important role in osteoclast differentiation upon RANKL stimulation and activates downstream NFκB family. CRE-Ter in a dose dependent manner reduced DNA binding activity of NFκB as revealed by EMSA. CRE-Ter had a profound influence on downregulation of miR-21 in osteoclast cultured after (RANKL) - induced murine monocyte/macrophage RAW 264.7 cells. Regulation of miR-21 by CRE-Ter required the activation of NFκB signaling pathway. NFκB is a transcription factor normally sequestered in the cytoplasm by the inhibitory protein IκBα. Phosphorylation and subsequent degradation of IκBα results in the release, activation and translocation of the subunit of NFκB into the nucleus, where they bind to target sequence on DNA and function as transcription factors. Here, our results have shown that CRE-Ter induced the degradation of IκBα and translocation of NFκB p65 subunit to the nuclease. The promoter region of miR-21 gene possesses two potential NFκB responsive elements, and direct binding of NFκB p65 protein to these elements has been reported in human biliary epithelial cells exposed to lipopolysaccharide [35]. This report showed that NFkB-Akt-miR-21 pathways could serve as an innovative target of therapeutics for bone resorptive conditions.

**Fig. 8:**
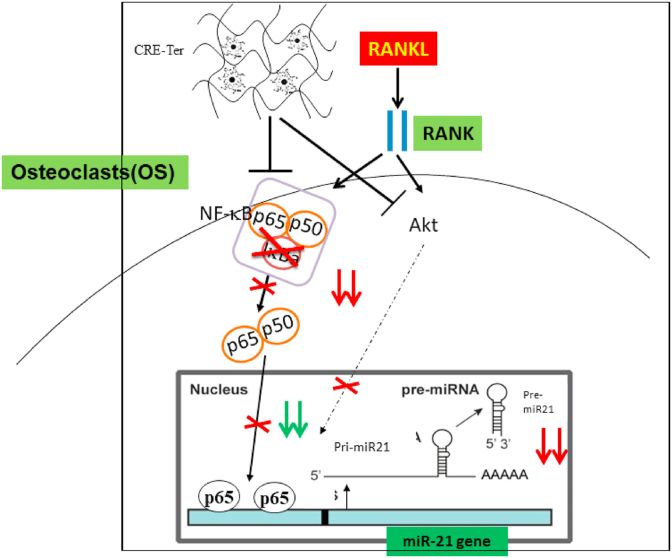
CRE-Ter has antiosteoclastogenic potential by reducing the *in vitro* RANKL induction of NFκB and Akt signaling pathways in osteoclast cells, and also by downregulation of miR-21 gene expression.

In summary, our findings clearly show that CRE-Ter has antiosteoclastogenic potential by reducing the *in vitro* RANKL induction of NFκB and Akt signaling pathways in osteoclast cells, and also by downregulation of miR-21 gene expression, one of the key signatures of osteoclastogenesis (Fig. 8). This study identifies CRE-Ter as a potent inhibitor of osteoclastogenesis and bone resorption and provides evidence of its therapeutic potential to treat diseases involving abnormal bone lysis.

## Declaration of competing interest

The authors declare that they have no conflicts of interest.
